# High prevalence of multiple antibiotic resistance in clinical *E. coli* isolates from Bangladesh and prediction of molecular resistance determinants using WGS of an XDR isolate

**DOI:** 10.1038/s41598-021-02251-w

**Published:** 2021-11-24

**Authors:** Preeti Jain, Asim Kumar Bepari, Prosengit Kumer Sen, Tanzir Rafe, Rashed Imtiaz, Maqsud Hossain, Hasan Mahmud Reza

**Affiliations:** 1 Department of Pharmaceutical Sciences, North South University, Dhaka, Bangladesh; 2 NSU Genome Research Institute (NGRI), North South University, Dhaka, Bangladesh; 3 Department of Biochemistry and Microbiology, North South University, Dhaka, Bangladesh

**Keywords:** Biochemistry, Biological techniques, Computational biology and bioinformatics, Genetics, Microbiology, Molecular biology, Pathogenesis

## Abstract

Multi-drug-resistance (MDR) is a severe public health concern worldwide, and its containment is more challenging in developing countries due to poor antimicrobial resistance (AMR) surveillance and irrational use of antibiotics. The current study investigated 100 clinical *E. coli* isolates and revealed that 98% of them were MDR. PCR analysis using 25 selected isolates showed the predominance of metallo-β-lactamase gene *bla*_NDM_ (80%) and ESBL genes *bla*_OXA_ (48%) and *bla*_CTX-M-15_ (32%). The AmpC gene was detected in 68% of the isolates, while 32% was *tet*C positive. Notably, 34% of the isolates were resistant to carbapenem. Whole genome sequence (WGS) analysis of an extensively drug-resistant (XDR) isolate (L16) revealed the presence of the notorious sequence type 131 responsible for multi-drug-resistant infections, multiple antibiotic resistance genes (ARGs), virulence genes, and mobile genetic elements that pose risks to environmental transmission. Our results indicate that MDR is alarmingly increasing in Bangladesh that critically limits the treatment option against infections and contributes to further aggravation to the prevailing situation of MDR worldwide. The findings of this study will be valuable in designing sustainable strategies to contain MDR in the region.

## Introduction

Bacterial resistance to antibiotics is a global health crisis with far-reaching consequences. By 2050, Drug-resistant infections may cause 10 million deaths annually, with approximately 90% of the predicted deaths to happen in Asia and Africa^1,2^. Indiscriminate use of antibiotics, inappropriate dosing, and incomplete treatment in both humans and animals are significant factors leading to the development of resistance in bacteria^3^. The situation is deteriorated further by the rapid spread of resistance genes. Several intrinsic factors such as point mutation, gene amplification, and extrinsic factors like horizontal transfer of resistance genes between bacteria within and across species by transposons, integrins, or plasmids have been suggested for the development of resistance, that cannot be restricted once developed even by restricting the antibiotic usage^4,5^. Significant rise in the presence of extended-spectrum beta-lactamases (ESBLs) such as TEM, SHV, OXA, CMY, and CTX-M, and AmpC beta-lactamases in recent years has made the situation alarming, along with the presence of carbapenem resistance that is mediated chiefly by *bla*_OXA_ and *bla*_NDM_ variants^3,6,7^. Understanding the antimicrobial resistance determinants in bacteria at the genetic level plays a critical role in controlling and understanding the ever-changing dynamics of resistance^8^.

On the other hand, continuous accumulation of multiple mutations leads to possible emergence of genomes resistant to antimicrobials^9^. Hence, whole-genome sequencing (WGS) of microorganisms has become a powerful approach for screening antibiotic resistance and evaluating the number of mutations and functions of the mutated genes^5,10^. WGS also holds great promise in developing newer antibiotics, controlling antimicrobial resistance, and enhancing diagnostics and public health microbiology^11,12^.

*Escherichia coli* (*E. coli*) is one of the major causes of nosocomial and community-associated infections, including respiratory tract infections (RTIs), urinary tract infections (UTIs), and enteric infections in Bangladesh^13,14^. The clinical threat posed by *E. coli* is mainly attributed to its ability to rapidly acquire antibiotic resistance through multiple mechanisms. Moreover, the emergence of *E. coli* strains with ESBL and AmpC β-lactamase carriers also render them resistant to other antibiotics such as aminogylcosides, fluororquinolones, tetracycline, chloramphenicol, sulfonamides, and carbapenems—the last resort in the treatment of many life-threatening infections^15^. *E. coli* is also considered a good indicator of antibiotic resistance in bacterial communities as it has been known to be a significant reservoir of genes coding for antimicrobial drug resistance^14^. Robust antibiotic resistance surveillance strategies combined with high-throughput tools such as WGS are common in developed countries. However, in resource-limited countries such as Bangladesh, data to monitor trends and susceptibility patterns against antibiotics are infrequently produced^2,16^. During the last decade, several studies conducted in Bangladesh revealed the irrational use of antimicrobials, prevalence of self-treatment, and incomplete therapy that contributed to the emergence of resistant bacterial strains^2,17,18^. Lack of systematic and detailed studies on antibiotic resistance patterns from Bangladesh often makes physicians prescribe multiple antibiotics, including broad-spectrum antibiotics, which further aggravates the AMR situation in Bangladesh, where infectious diseases still hold the highest morbidity and mortality rate^18,19^.

In this study, we determined the antibiotic resistance pattern in *E. coli* isolates from clinical urine and sputum specimens collected from pathological laboratories of Dhaka, Bangladesh. The study also screened the ESBL genes (TEM, CTX-M-15, SHV, and OXA), AmpC, tetracycline, and carbapenem resistance genes such as OXA-47 and NDM in MDR isolates. We also report the whole genome characterization of an XDR *E. coli* strain isolated from a urine sample. In order to understand the phylogenomic changes, presence of genetic elements, and virulence, a whole-genome analysis of the isolate, including its plasmids and comparative multilocus sequence typing (MLST), was carried out. The concordance between WGS-based AMR prediction and the phenotype identified from conventional antimicrobial susceptibility tests was also evaluated.

## Results

**Figure 1:**
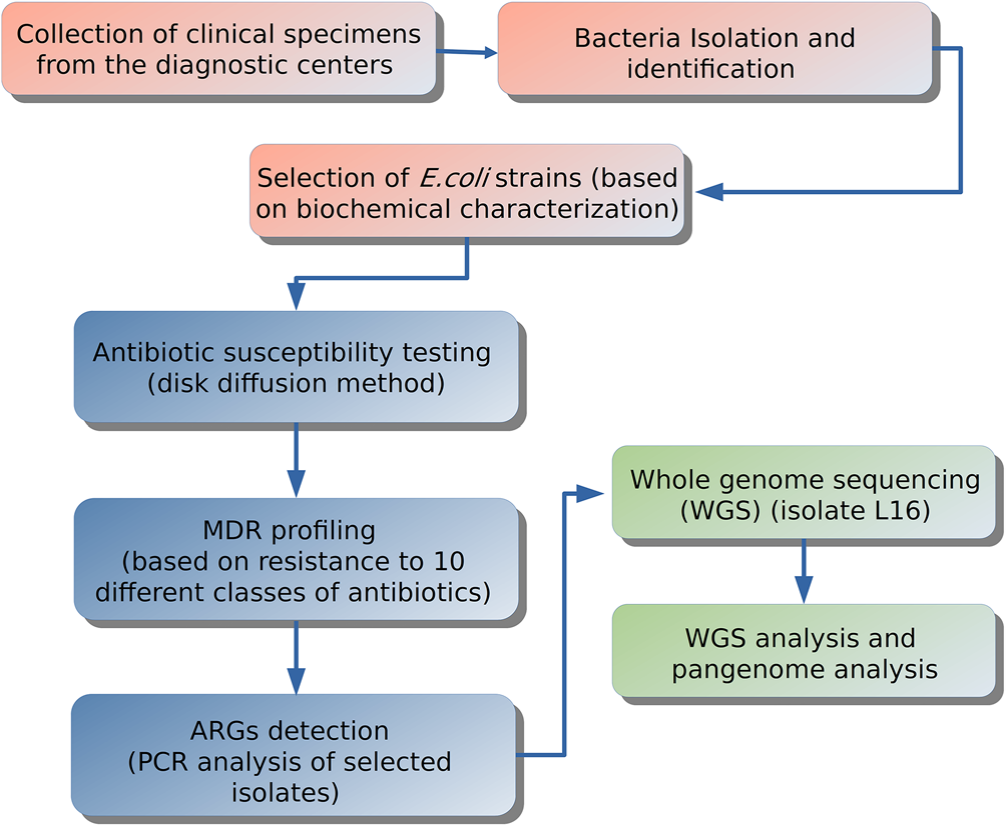
Schematic illustration of workflow of the entire procedure and analysis.

The current study has been conducted on 100 *E. coli* isolates obtained from clinical specimens collected from different diagnostic centers in Dhaka, Bangladesh. Isolates were subjected to antibiotic susceptibility testing after biochemical characterization. This was followed by PCR-based detection for ARGs and WGS analysis. A schematic illustration of the workflow of the entire procedure and analysis is shown in Fig. 1.

### Bacterial isolates and biochemical characterization

A total of 100 *E. coli* isolates were isolated from 100 clinical samples, which consisted of 90% urine and 10% sputum specimens. Based on gram-staining, morphological features, culture characteristics, and biochemical characterization, the isolates were confirmed as *E. coli*. All *E. coli* isolates appeared bright pink on MacConkey agar, and the colonies showed a characteristic metallic green sheen when cultured on EMB agar. All isolates were gram-negative and fermented dextrose, maltose, lactose, sucrose, and mannitol with acid and gas production. Catalase, Methyl red, and indole test of the *E. coli* isolates showed positive results, but the V-P test was negative.

### Antimicrobial resistance profile

An antibiotic susceptibility test was carried out on all the 100 isolates against 12 different antibiotics. As shown in Table 1, the highest percentage of resistance was observed against amoxicillin (98%), followed by cefuroxime (75%) and cotrimoxazole (62%). Sixty percent of the *E. coli* isolates were resistant to piperacillin-tazobactam, a beta-lactam/beta-lactamase inhibitor combination. Resistance to gentamicin, ciprofloxacin, norfloxacin, chloramphenicol, and azithromycin varied between 34% and 49%. A significantly high degree of resistance was observed to carabapenems, 30% to meropenem and 38% to imipenem.

**Table 1:** Antibiotic susceptibility patterns of *E. coli* isolates (n = 100).

Antibiotic	Resistance%	Intermediate%	Sensitive%
**Penicillins**			
Amoxicillin (10 μg)	98	2	
**Aminoglycosides**			
Gentamicin (10 μg)	39	13	48
**Fluoroquinolones**			
Ciprofloxacin (5 µg)	34	8	58
Norfloxacin (10 μg)	39	5	56
**Cephalosporins**			
Cefuroxime (30 μg)	75	5	20
**Carbapenems**			
Imipenem (10 μg)	38	9	53
Meropenem (10 μg)	30	8	62
**Phenicols**			
Chloramphenicol (30 μg)	40	12	48
**Macrolides**			
Azithromycin (15 μg)	49	17	34
**Tetracyclines**			
Tetracycline (30 μg)	55	12	33
**Sulfonamides**			
Cotrimoxazole (25 μg)	62	11	27
**Penicillins with β-lactamase inhibitors (Beta-lactam/beta-lactamase inhibitor)**			
Piperacillin-tazobactam (100/10 μg)	60		40

*n* number of isolates.

### Prevalence of multiple drug resistance

**Figure 2:**
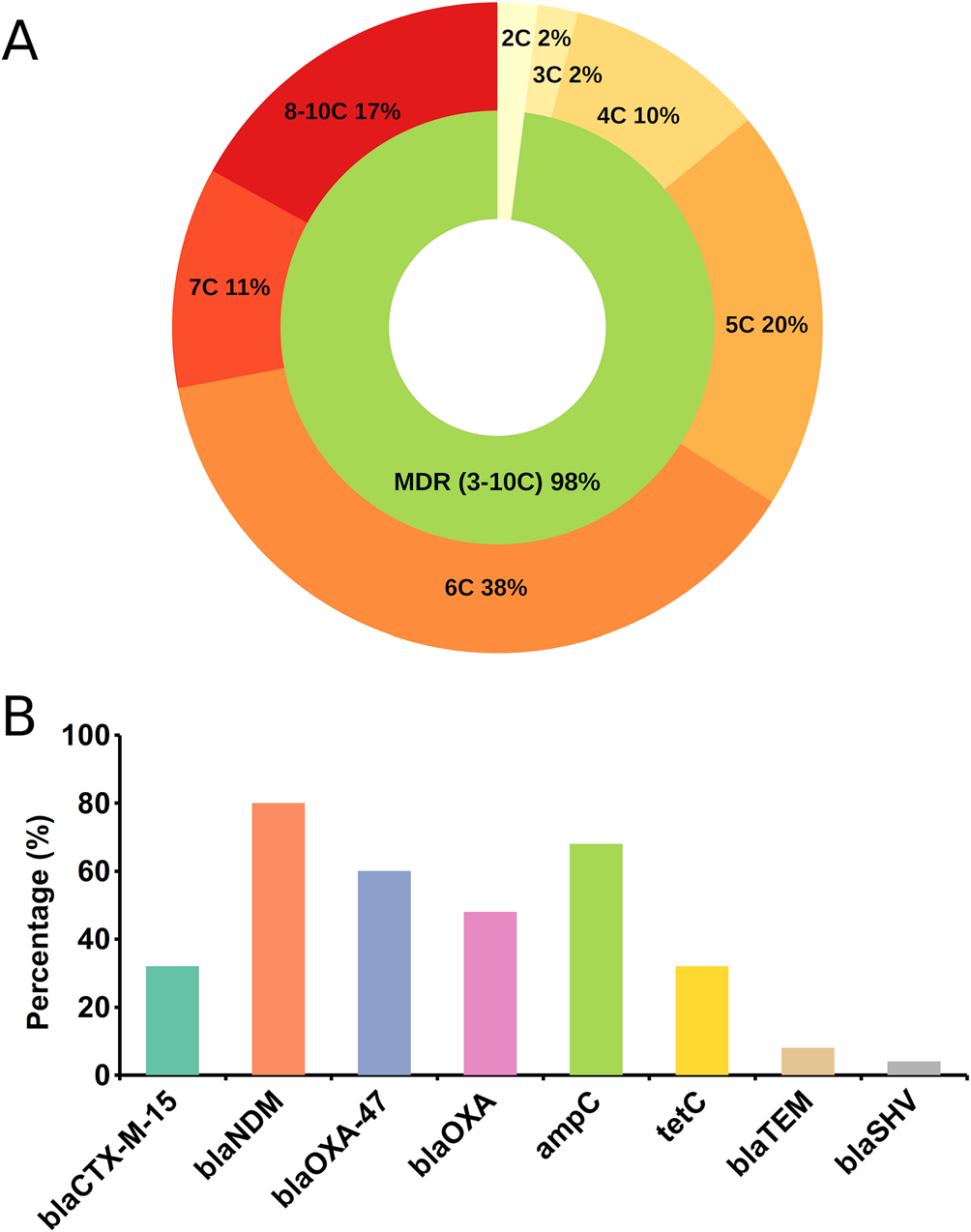
Prevalence of multiple antibiotic resistance among the isolates. (**A**) Distribution of resistance pattern against different classes of antibiotics. (**B**) Prevalence of ARGs among the tested *E. coli* isolates. C, classes.

In this study, 96% of isolates showed a MAR index greater than 0.3, whereas 64% showed a MAR index above 0.5. Figure 2A shows a high prevalence of multiple antibiotic resistance among the isolates where 98% of the isolates were MDR, and the highest frequency of resistance to multiple antibiotics classes observed was 38.78%, with resistance to six different classes of antibiotics. Moreover, 16% of the isolates showed extensive drug resistance (XDR), with one of the isolates being resistant to all the antibiotics used in this study.

### Detection of antibiotic resistance genes

Based on the antibiogram data, 25 isolates were studied for the presence of antibiotic resistance genes. The overall prevalence of ARGs among the investigated *E. coli* isolates with their resistance phenotype is shown in Table 2. All the isolates were positive for at least one AMR gene. The most prevalent gene was metallo-β-lactamase *bla*_NDM_ harbored by 80% of the isolates, which confers resistance to carbapenems. Another gene, *bla*_OXA-47_, which confers resistance to carbapenems, was present in 60% of the isolates. A total of 17 (68%) isolates were AmpC-positive, while tetC gene was detected in 8 (32%) isolates. Among the ESBL genes, *bla*_CTX-M-15_, *bla*_TEM_ and *bla*_OXA_ were detected in 32%, 8%, 48% of the isolates, respectively, while only one isolate (4%) exhibited the presence of *bla*_SHV_. The prevalence of different ARGs is shown in Fig. 2B.

**Table 2:** Phenotypic antibiotic resistance and distribution of resistance genes among *E. coli* isolates.

Isolate	Resistance phenotype	Resistance ESBL genes including carbapenemnase	AmpC and tetC genes
GSU-1	Amx, Gtm, Cip, Nor, Cxm, Imp, Mem, Azm, Te, Ctm, Ptz	*bla*_NDM,_ *bla*_OXA-47_	*ampC*
GSU-2	Amx, Cxm, Imp, C, Te, Ctm	*bla*_OXA-47_	
GSU-3	Amx, Cxm, Imp, Azm, Te, Ctm	*bla*_OXA,_ *bla*_CTX,_ *bla*_OXA-47_	*ampC*
GSU-4	Amx, Nor, Cxm, Imp, Ctm, Ptz	*bla*_NDM_	
GSU-5 (L16)	Amx, Gtm, Cip, Nor, Cxm, Imp, Mem, C, Azm, Te, Ctm, Ptz	*bla*_TEM_, *bla*_CTX_, *bla*_OXA_, *bla*_OXA-47_, *bla*_NDM_	*ampC, tetC*
GSU-6	Amx, Gtm, Cip, Nor, Cxm, Imp, Azm, Te, Ctm	*bla*_OXA-47_, *bla*_NDM_	*ampC, tetC*
GSU-7	Amx, Cxm, Imp, C, Azm, Te	*bla*_CTX_, *bla*_OXA_, *bla*_NDM_	*ampC*
GSU-8	Amx, Nor, Cxm, Imp, Azm, Te, Ctm, Ptz	*bla*_OXA_, *bla*_SHV_, *bla*_NDM_, *bla*_OXA-47_	*ampC, tetC*
GSU-9	Amx, Cip, Cxm, Imp, Azm, Te, Ctm, Ptz	*bla*_NDM_	*ampC*
GSU-10	Amx, Gtm, Nor, Cxm, Imp, Mem, Azm, Ctm, Te, Ptz	*bla*_OXA_, *bla*_NDM_, *bla*_OXA-47_	
GSU-11	Amx, Cip, Nor, Cxm, Imp, Mem, Ctm, Ptz	*bla*_CTX_, *bla*_NDM_, *bla*_OXA-47_	*ampC*
GSU-12	Amx, Cip, Nor, Cxm, Imp, Mem, Azm, Ptz	*bla*_CTX_, *bla*_NDM_	*ampC, tetC*
GSU-13	Amx, Cip, Nor, Cxm, Imp, Azm, Te, Ctm, Ptz	*bla*_OXA_, *bla*_TEM_, *bla*_NDM_, *bla*_OXA-47_	*ampC, tetC*
GSU-14	Amx, Cip, Nor, Cxm, Imp, C, Azm,Te, Ctm, Ptz	*bla*_OXA_, *bla*_NDM_	*tetC*
GSU-15	Amx, Nor, Cxm, Imp, Ptz	*bla*_OXA_	
GSU-16	Amx, Gtm, Cip, Nor, Cxm, Imp, C, Azm,Ctm, Ptz	*bla*_OXA-47_, *bla*_NDM_	*ampC*
GSU-17	Amx, Gtm, Nor, Cxm, Imp, C, Ptz	*bla*_OXA_, *bla*_NDM_	*ampC*
GSU-18	Amx, Gtm, Nor, Cxm, Imp, Azm, Ctm, Ptz	*bla*_OXA_, *bla*_*NDM*_	*ampC*
GSU-19	Amx, Gtm, Cxm, Imp, Azm, Ctm,	*bla*_NDM_	*ampC*
GSU-20	Amx,Nor, Cxm, Imp, C, Azm,Te,Ctm,Ptz	*bla*_OXA-47_, *bla*_NDM_	*ampC, tetC*
GSU-21	Amx, Nor, Cxm, Imp, Azm,	*bla*_OXA-47_	
GSU-22	Amx, Cip, Cxm, Imp,Ptz	*bla*_OXA_	
GSS-23	Amx, Gtm, Nor, Cxm, Imp, Mem, C, Ctm, Ptz	*bla*_CTX_, *bla*_OXA_, *bla*_OXA-47_, *bla*_NDM_	*ampC*
GSS-24	Amx, Gtm, Cip, Nor, Cxm, Imp, Mem,Te, Ctm, Ptz	*bla*_CTX_, *bla*_NDM_, *bla*_OXA-47_	
GSS-25	Amx, Gtm, Cip, Nor, Cxm, Imp, C, Te, Ctm, Ptz	*bla*_CTX_, *bla*_OXA-47_, *bla*_NDM_	*ampC, tetC*

*Amx* amoxicillin, *Cip* ciprofloxacin, *Gtm* gentamycin, *Imp* imipenem, *Mem* meropenem, *Nor* norfloxacin, *C* Chloramphenicol, *Te* tetracycline, *Ptz* piperacillin & tazobactam, *Cxm* cefuroxime, *Ctm* cotrimoxazole, *Azm* azithromycin.

### Whole-genome sequencing

We performed a paired-end WGS of the bacterial isolate showing the highest antimicrobial resistance. Sequencing analysis revealed that the quality of both the forward and the reverse reads was considerably high. There were approximately 2.8 million sequences, and no read had poor quality. Duplicate counts were 29% and 18.5%, and GC contents were 50% and 51% for the forward and reverse reads, respectively. For most reads, the mean quality values (Phred scores) across different base positions were greater than 30 (Fig. S1).

### Genome assembly and annotation

The reads were subjected to the comprehensive genome analysis service at PATRIC^20^ where the strain was designated as *Escherichia coli* Strain-L16 (L16). This genome appears to be of good quality based on the annotation statistics and comparing other genomes in PATRIC within this same species. L16 reads were trimmed and assembled using unicycler, a de novo genome assembly tool^21^. There were 118 contigs, an estimated genome length of 5,137,608 bp, and an average GC content of 50.61%. The N50 length, defined as the shortest sequence length at 50% of the genome, is 225,781 bp. The L50 count, which is defined as the smallest number of contigs whose length sum produces N50, is 8. The lengths of the smallest and the largest contigs were 511 bp and 673,523 bp, respectively.

The L16 genome was annotated using the RAST tool kit (RASTtk)^22^ and assigned a unique genome identifier (562.73579) in PATRIC. This genome is in the superkingdom Bacteria and was annotated using genetic code 11. The taxonomy of this genome is: cellular organisms > Bacteria > Proteobacteria > Gammaproteobacteria > Enterobacterales > Enterobacteriaceae > Escherichia > *Escherichia coli*. This genome has 5222 protein-coding sequences (CDS), 79 transfer RNA (tRNA) genes, and four ribosomal RNA (rRNA) genes. The annotation included 593 hypothetical proteins and 4629 proteins with functional assignments. The functional assignments included 1319 proteins with Enzyme Commission (EC) numbers, 1098 with Gene Ontology (GO) assignments, and 925 proteins mapped to KEGG pathways. PATRIC annotation includes two types of protein families, and this genome has 5004 proteins belonging to the genus-specific protein families (PLFams), and 5088 proteins belong to the cross-genus protein families (PGFams). Figure S2 shows a circular representation of the L16 annotation features.

A subsystem is a set of proteins that implement a specific biological process or structural complex, and PATRIC annotation includes an analysis of the subsystems unique to each genome^23^. We compared the subsystems of L16 with two *E. coli* reference genomes, K-12 substr. MG1655 and O157:H7 str. Sakai (Fig. S3). K-12 substr. MG1655 is a nonpathogenic strain, whereas O157:H7 str. Sakai is a pathogenic strain. Overall, the subsystems of L16 are very similar to those of two reference genomes. The number of genes annotated in the subsystem superclasses of cellular processes, energy, membrane transport, and metabolism was slightly higher in the genome L16.

Many annotated genes in L16 have homology with known genes for antibiotic resistance, drug targets, transporters, and virulence factors. Table S1 provides a comparison of annotated gene numbers among L16 and two reference genomes.

### In silico typing

Based on the allelic combination of the seven genes, isolate L16 was identified as having the sequence type (ST) 131. ST131 is a highly virulent pathogenic clone^24^ of the subgroup extraintestinal pathogenic *E. coli* (ExPEC)^25^.

Based on the wzx and wzy genes encoding cell surface O antigens and the fliC gene encoding the flagellar antigen H, L16 was identified as serotype O25:H4. In silico phylotyping predicted phylogroup B2 for the L16 genome.

### Antimicrobial resistance genes

Annotated AMR genes in L16 are listed in Table 3, which shows that majority of them are involved in conferring resistance via efflux pumps and modified antibiotic targets.

**Table 3:** AMR genes of L16 annotated in PATRIC.

AMR mechanism	Genes
Antibiotic activation enzyme	*KatG*
Antibiotic inactivation enzyme	*AAC(3)-II,III,IV,VI,VIII,IX,X, BlaEC* family*, CatB* family, *CTX-M* family, *OXA-1* family
Antibiotic resistance gene cluster, cassette, or operon	*MarA, MarB, MarR*
Antibiotic target in susceptible species	*Alr, Ddl, dxr, EF-G, EF-Tu, folA, Dfr, folP, gyrA, gyrB, inhA, fabI, Iso-tRNA, kasA, MurA, rho, rpoB, rpoC, S10p, S12p*
Antibiotic target protection protein	*BcrC*
Efflux pump conferring antibiotic resistance	*AcrAB-TolC, AcrAD-TolC, AcrEF-TolC, AcrZ, EmrAB-TolC, EmrD, EmrE, EmrKY-TolC, MacA, MacB, MdfA/Cmr, MdtABC-TolC, MdtEF-TolC, MdtL, MdtM, SugE, TolC/OpmH*
Gene conferring resistance via absence	*gidB*
Protein altering cell wall charge conferring antibiotic resistance	*GdpD, PgsA*
Regulator modulating expression of antibiotic resistance genes	*AcrAB-TolC, EmrAB-TolC, GadE, H-NS, OxyR*

Analysis of L16 resistome using the RGI tool as mentioned in methods identified 8 perfect hits and 53 strict hits (Supplementary Dataset 1).

Notably, the presence of the CTX-M-15 allele renders the *E. coli* clone resistant to extended-spectrum-beta-lactamases (ESBLs). The L16 genome is positive for several genes and mutations, such as mdtH, emrA, emrB, emrR, mutated gyrA, and mutated parC, conferring fluoroquinolone resistance. Moreover, this clone also has several multidrug-resistant genes including acrB, AcrS, cpxA, evgA, hns, gadW, TolC, mdtM, mdtF, and marA. Together, the presence of multiple resistant genes is expected to make the strain resistant to aminoglycosides, carbapenem, cephalosporins, cephamycin, fluoroquinolones, macrolides, penicillin, and tetracyclines (Table 3, Supplementary Dataset 1).

### Virulence factor genes

In PATRIC, L16 was annotated for 95 genes with homology to genes of the VFDB (Supplementary Dataset 2). There are 35 genes with iron uptake functions and 28 genes with adherence functions. Sixty of these genes were predicted to be of *E. coli* origin, whereas 35 genes originated in other species.

### Mobile genetic elements

In the L16 genome, 14 mobile genetic elements (MGEs) were predicted using the MobileElementFinder web tool^26^. Three of the MGEs are miniature inverted repeats, and the remaining 11 are insertion sequences (Table 4). MITEEc1 is an IS630 family MGE (https://www-is.biotoul.fr/) and an Enterobacterial repetitive intergenic consensus (ERIC) sequence with possible functions in mRNA stability^27^. In the L16 genome, one copy of MITEEc1 is located in contig 1 between the genes YbhJ (encodes a conitase family protein) and YbhI (encodes an inner membrane protein). Contig 10 of L16 has a 2410 bp-log IS21 family transposase. The longest MGE predicted in L16 was the IS682, an IS66 family MGE consisting of three open reading frames (ORFs) including two accessory genes TnpA, TnpB, and a DDE transposase element (https://www-is.biotoul.fr/).

**Table 4:** Mobile genetic elements (MGEs) in the L16 genome.

Name	Type	Allele_length	E_value	Identity (%)	Coverage (%)
MITEEc1	Miniature inverted repeat	123	2.3E−49	96.7	100.0
ISKpn8	Insertion sequence	1443	0	94.7	100.0
MITEEc1	Miniature inverted repeat	123	2.0E−36	90.2	100.0
ISEc10	Insertion sequence	2410	0	100.0	100.0
MITEEc1	Miniature inverted repeat	122	8.0E−53	98.4	99.2
ISEc1	Insertion sequence	1291	0	96.6	100.0
ISEc53	Insertion sequence	1885	0	100.0	100.0
ISEc38	Insertion sequence	1722	0	95.2	100.0
IS30	Insertion sequence	1221	0	99.8	100.0
IS629	Insertion sequence	1310	0	94.7	100.0
ISKpn37	Insertion sequence	1262	0	97.1	99.3
IS629	Insertion sequence	1310	0	94.1	100.0
IS682	Insertion sequence	2532	0	90.9	99.6
IS26	Insertion sequence	820	0	100.0	100.0

### Pangenome analysis

**Figure 3:**
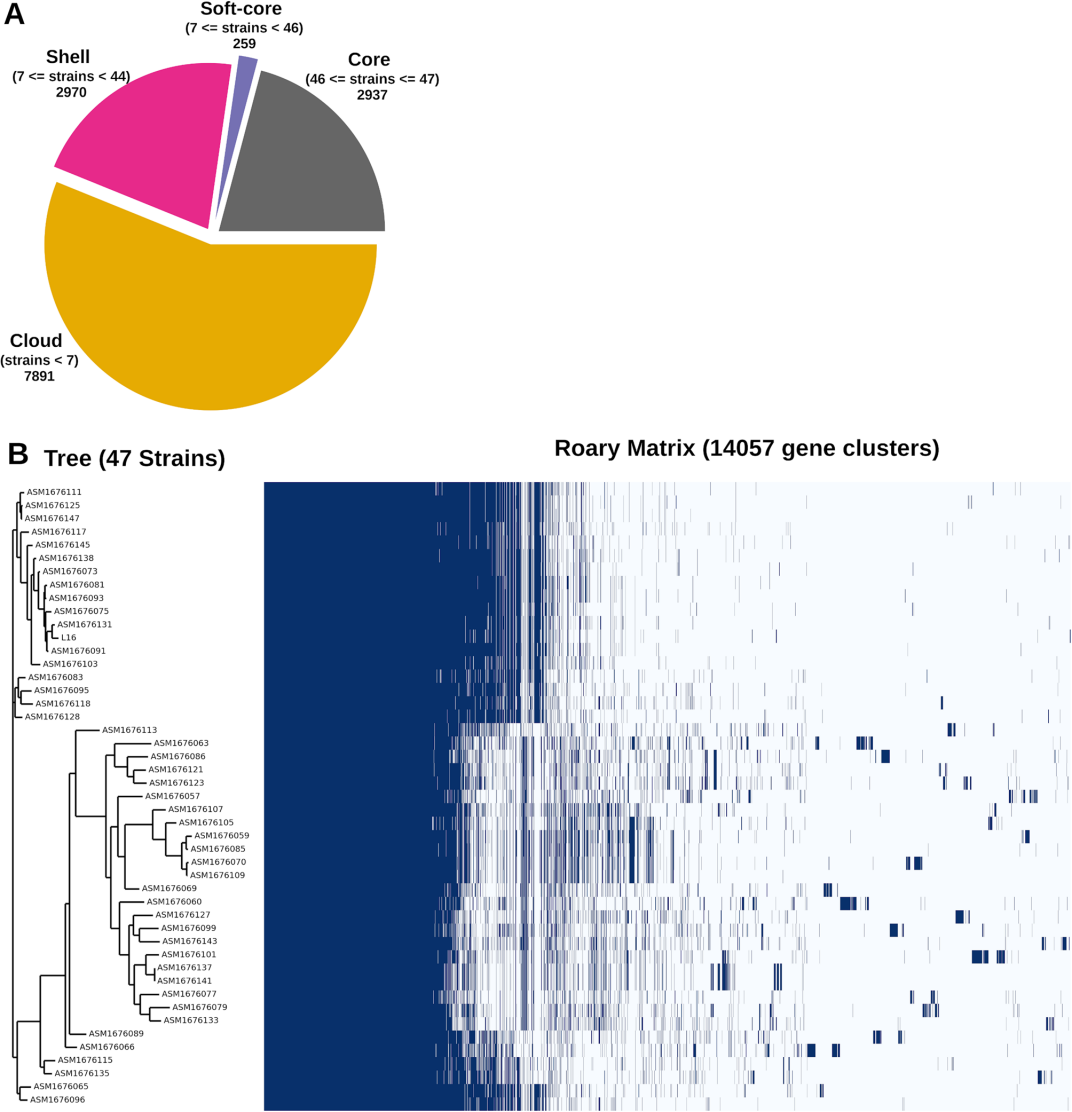
Pangenome analysis of L16 and 46 other strains of Bangladeshi origin. (**A**) Number of genes in the pangenome. (**B**) Roary matrix shows clustering of genes among 47 strains.

*E. coli* harbors an open pangenome that is evolving continuously. Rasko et al. determined a reservoir of more than 13,000 *E. coli* genes in 2008^28^. We performed pangenomic analysis of L16 and 46 genome assemblies of Bangladeshi origin from NCBI (https://www.ncbi.nlm.nih.gov/assembly). The pangenome consisted of 14,057 genes, of which 2937 are in the core (Fig. 3A). The pangenome matrix shows clusters of genes and dendrogram of the isolates (Fig. 3B). With 13 other isolates, L16 is in a cluster where more genes are in the core than two other clusters. The isolate ASM1676131 is the closest relative in the dendrogram. Notably, all but one genome (ASM1676075v1) are ST131 clones. Analyzing the gene presence-absence, we found 26 unique genes (Table S2) in L16. The L16 genome has at least 90 prophages, including four unique prophages listed in Table S2.

**Figure 4:**
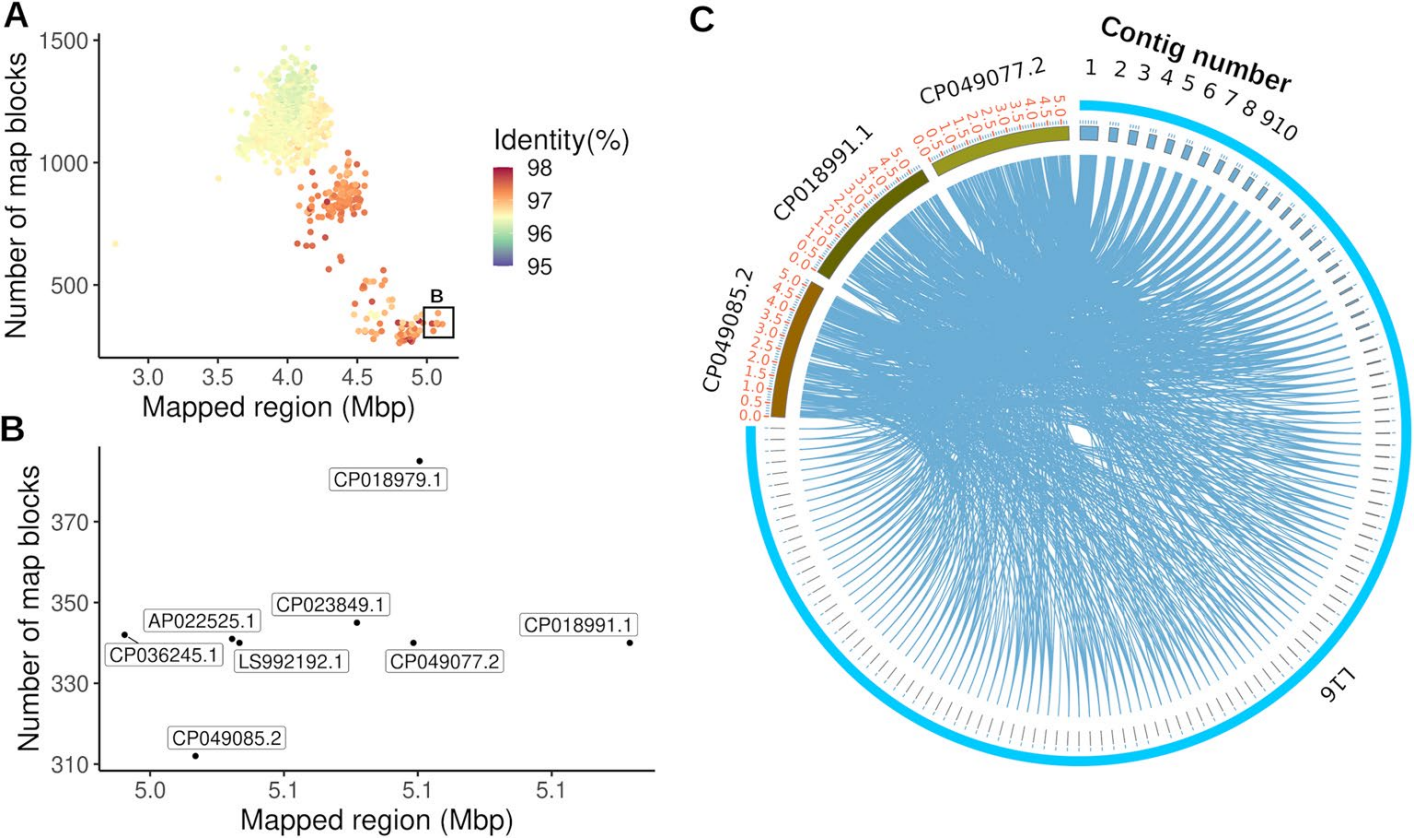
Mapping of L16 genome to other public *E. coli* genomes. (**A**) Mapping of L16 genome to all complete public *E. coli* genomes. Inset shows eight genomes with longest mapped regions. (**B**) Mapping to eight closely related genomes (amplified view of the inset in (**A**). (**C**) Circos presentation of L16 mapping to three closest public genomes.

Considering the global dissemination of the *E. coli* ST131, we next explored the relatedness of the L16 genome to all publicly available complete *E. coli* genomes. We downloaded 1253 assemblies from NCBI (https://www.ncbi.nlm.nih.gov/assembly, accessed on April 11, 2021) and mapped the L16 genome to each of the public *E. coli* genomes using lastz^29^. After filtering out the hits below 90% match, average percent identity and total mapped sequences were tabulated for each chromosome from the assemblies and plotted on a scatter chart (Fig. 4A). Except for one outlier, all public sequences matched with 3.5–5.1 Mbp of the L16 genome. The eight genomes showing the highest identity are shown in Fig. 4B. These isolates were collected between 2009 and 2018 from Australia, Germany, Italy, Japan, Sweden, and the USA (Table S3).

From lazt mapping, we identified three public *E. coli* genomes with the highest mapped sequences for L16 and visualized the mapping in a circos plot (Fig. 4C). Apparently, the L16 genome is almost identical to those public genomes (Fig. 4C). Next, we aligned the AMR protein features of L16 to the eight closely related genomes (Fig. S4). As expected, the features were almost identical among the genomes except that L16 carries the chloramphenicol O-acetyltransferase (Fig. S4).

## Discussion

Among all the WHO regions, southeast Asian countries including Bangladesh are at the highest risk of AMR^18^, and ESBL-producing carbapenem-resistant *E. coli* are among the global priority pathogens list of WHO categorized as critical^30^. The strategies to contain AMR rely on a robust antibiotic surveillance system, assessing the threats imposed due to the emergence of MDR and implementation of the findings. Scarcity of data on AMR patterns and minimal information on the presence of ARGs and molecular determinants of resistance prompted this study in which we investigated resistance phenotypes among 100 *E. coli* isolates collected from clinical specimens obtained from diagnostic centers in Dhaka, Bangladesh.

In the current investigation, 98% of isolates were MDR, defined as resistant to three or more antibiotics classes, which is alarming (Fig. 2A). The highest resistance was observed against amoxicillin (98%) which is more or less similar to recent study reports from Bangladesh^13,18,31^. We observed higher resistance to cefuroxime (75%) compared to 53. 81% as reported by Nahar et al. in 2017^31^, which could be attributed to the sampling area. Among the antimicrobials tested, imipenem, ciprofloxacin, norfloxacin, and meropenem were found as the most effective agents against *E. coli* in this study. However, the rate of resistance against carbapenem was much higher (> 30%) in comparison to recent reports involving *E. coli* isolates from clinical specimens by Nahar et al. 2017 (< 1.5%)^31^, Hosaain et al. 2021 (< 3%)^32^ and Nobel et al. 2021 (< 10%)^13^ from Bangladesh. A study from India reported the presence of 29.54% carbapenem resistance in *E. coli* isolates^33^, which is in line with the current study. However, some studies from India and Nepal^34,35^ reported much higher resistance to meropenem (60–75%). Carbapenems are considered the last shelter for gram-negative bacteria treatment, but carbapenem resistance is increasing in Southeast Asian countries, which is dangerous. Resistance observed against gentamicin (39%) and cotrimoxazole (62%) in this study is consistent with the findings of Nahar et al.^31^. However, some studies from Bangladesh showed lesser resistance to gentamicin (< 15%), cotrimoxazole (< 50%), and also to tetracycline (34%)^32,36^. These variations could be attributed to the prevailing usage and drug abuse in the study area, which warrants stringent and continuous surveillance on antibiotic resistance patterns. Resistance to other drugs are in agreement with some previously published reports^31,36^ with some minor variations. Sixty four percent of the isolates showed MAR index greater than 0.5, which indicates a very high prevalence of multiple drug resistance among clinical *E. coli* isolates in Bangladesh. Out of the 100 isolates, 38.78% showed resistance to 6 out of 10 classes of antibiotics tested, and 16% of isolates were XDR. Although the number of isolates from sputum samples (10) was smaller than urine samples (90), similar or higher resistance was observed against all the antibiotics except cefuroxime, meropenem, and tetracycline. The percentage of isolates from urine with a MAR index greater than 0.3 was higher (96.67%) than sputum isolates (90%). Reports emanating from developing countries like Bangladesh indicate a high prevalence of multidrug-resistant *E. coli* in clinical as well as in community settings such as drinking water and poultry^2,3,14^. However, limited information is available on the prevalence and the genotypic characteristics of antibiotic-resistant strains associated with different ecological niches in Bangladesh^3,14^. The widespread availability of antimicrobials without prescription in the country, self-medication, non-compliance of dosage, and irrational use across agriculture, poultry and health sectors have contributed significantly to the spread of resistant strains in Bangladesh^18^. The presence of antimicrobial residues such as tetracycline, ciprofloxacin, and amoxicillin have been observed in a high percentage of poultry meat and eggs^18,37^. Discharge of untreated medical wastes resulting in the presence of high levels of resistant *E. coli* in the water has also been reported^38^. This implies co-selection of resistance towards multiple classes of antibiotics leading to the rapid dissemination of MDR in same or distantly related species, which could be the greatest threat to public health in the twenty-first century. The prevalence of ARGs is the major contributor to the development of multidrug and pandrug-resistant bacteria. However, data on the prevalence of antibiotic resistance genes in clinical *E. coli* isolates and the molecular basis of resistance is very limited in Bangladesh^2^. In this study, *bla*_NDM_ was predominant (80%) among the eight tested antibiotic resistance genes. Among metallo-β-lactamases (MBLs), NDM-1 has received wide attention because of its broad hydrolytic activity on β-lactam antibiotics, including carbapenems, and carriage of NDM-1 enables drug resistance to move between communities and hospitals. The prevalence of *bla*_NDM-1_ has been previously reported in several studies^39,40^. Safain et al. reported NDM-1 (55%) as the most prevalent resistance gene in Bangladesh^2^. In the current study, we found that 80% of the isolates harbored *bla*_NDM-1_, which is alarming as therapeutic options against NDM-1 producers are rare. NDM-producers have been reported to be on the rise in India, Pakistan, and Nepal, indicating inappropriate and non-prescription antibiotic use as a probable cause of the development of resistance in this subcontinent^2,6,35^. Apart from NDM, other genes frequently associated with carbapenem resistance are *bla*_OXA-47_ and *bla*_OXA-1_, which were detected in 60% and 48% of the isolates. These findings are in accordance with the high phenotypic resistance observed against carbapenems (Tables 1, 2). The *bla*_OXA-1_ gene has frequently been associated with genes encoding extended-spectrum β-lactamases (ESBLs) and suggested imparting resistance to penicillin/inhibitor combinations^41^. Out of the 25 isolates tested for ARGs, 15 were resistant to piperacillin/tazobactam and ten harbored the *bla*_OXA-1_ gene (67%) (Tables 1, 2) which is approximately three times higher than that reported by Livermore et al. (12/59)^41^. Co-occurrences of *bla*_NDM_ along with variants of *bla*_OXA_ have been reported previously^42^. Similar results were observed in our study with the presence of *bla*_NDM_ + *bla*_OXA-47_ and *bla*_NDM_ + *bla*_OXA-1_ combinations in 12 and 9 isolates, respectively, while 5 isolates showed co-occurrence of *bla*_NDM_ along with *bla*_OXA-1_ and *bla*_OXA-47_ genes. Frequent association of *bla*_OXA-1_ with the CTX-M-15, the most abundant ESBL determinant in human *E. coli* isolates, has been reported from diverse geographical origins, contributing to resistance towards beta-lactam lactamase inhibitor combinations^42^. Among the isolates tested for ARGs, 32% showed the presence of *bla*_CTX-M-15_. A recent study reported a higher prevalence of *bla*_CTX-M-15_ (52%)^43^ among ESBL-producing *E. coli* isolates, causing extraintestinal infections in Bangladesh. A study from Egypt reported dominance of CTX-M-15 (87%) among CTX-M genotypes^44^. Isolates producing β-lactamases such as CTX-M-15 are associated with clonal lineages such as ST131, which spread extensively via MGEs, leading to a rapid increase in the prevalence of urinary tract and bloodstream infections worldwide^43,45^. The other most frequently described enzymes in *E. coli* include TEM and SHV types in which point mutations are suggested to give rise to ESBLs^44^. Although the abundant presence of *bla*_TEM_ has been described in several studies, including some from Bangladesh, the current study revealed the presence of *bla*_TEM_ only in 8% of the isolates, which is lower than a previously published report from Bangladesh^46^. The presence of *bla*_SHV_ was least in this study (4%) which is in line with the findings of Ahsan and Islam^46^. As the representatives of efflux mechanism of tetracycline, t*et*A and *tet*C genes are predominant in *E. coli*^47^. In this study, we determined the presence of *tet*C, which is higher (32%) than some previous studies^48,49^, indicating increased resistance to tetracycline. AmpC β-lactamases are a major clinical concern. They confer resistance to a wide range of β-lactam drugs, including penicillins, cephamycin, 1st-3rd generation cephalosporins, and classical β-lactamase inhibitors like clavulanic acid and tazobactam^50,51^. The expression level of the AmpC gene could be changed due to mutations in the promoter region which implies that phenotypic resistance detection against beta-lactam antibiotics^52,53^ is vital for clinical interventions. In this study, all the AmpC positive isolates showed 100% resistance to three (amoxicillin, imipenem, and cefuroxime) out of five beta-lactam antibiotics tested. In contrast, resistance against piperacillin-tazobactam and imipenem was observed in 76.47% and 29.41% of the isolates, respectively. Also, the AmpC gene was the second most prevalent among the ARGs, with 68% of the tested isolates being positive for AmpC.

The higher prevalence of AmpC beta-lactamases is also reported by Satter et al., 2020 from Bangladesh^54^. However, Ahsan and Islam reported a much lower prevalence (6%)^46^. This calls for continuous surveillance studies to fill the gaps in knowledge regarding the prevalence of ARGs. In Asia, the percentage of amp C positive clinical *E. coli* isolates varies over a wider range^55^, with the highest prevalence in India and Nepal^35,56^.

In the present study, we investigated the genome of an extensively drug-resistant isolate designated as L16 that showed resistance against all the antibiotics and presence of all the ARGs tested except SHV to determine the molecular mechanisms of resistance. L16 also showed the presence of multiple resistant genes (Table 3) to make the strain resistant to aminoglycosides, carbapenem, cephalosporins, cephamycin, fluoroquinolones, macrolides, penicillin, and tetracyclines (Table 3, Supplementary Dataset 1). These findings strongly support the AMR phenotype of L16 determined by antibiotic susceptibility test as well as PCR assay (Table 2). Many annotated genes in L16 showed homology with known genes for antibiotic resistance, drug targets, transporters, and virulence factors. MLST, one of the gold standards for determining epidemiological relatedness of organisms, showed that the isolate L16 belonged to the sequence type (ST) 131. ST131 has been notorious for causing worldwide pandemic multidrug-resistant infections crossing borders of countries, communities, and hospitals^57^. A serious concern for public health, the ST131 clones show broad resistance to extended-spectrum beta-lactams, fluoroquinolones, and also to carbapenems^6,57^. The isolate L16 belonged to phylogroup B2, which is predominant in blood and urinary tract infections that have a poor prognosis^58^ and also related to inflammatory bowel disease (IBD)^59^. L16 was annotated for 95 genes with homology to virulence genes of the VFDB (Supplementary Dataset 2), which shows the pathogenicity of the clone. The extreme pathogenicity of ST131 clones has been attributed to the presence of a large number of virulence factors, in addition to the appearance of multidrug-resistant genes^24^. The study of mobile genetic elements showed transposase ISEc10 apart from other MGEs, which might lead to overproduction of the AmpC-type beta-lactamase^60^. This also supports the findings from the PCR assay. Also, the longest MGE predicted in L16 was the IS682, which belongs to the IS66 family that is usually present in gram-negative rods and cocci^61,62^, and an association has been proposed between the presence of *Helicobacter pylori* (*H. pylori*) genes TnpA and TnpB and development of gastrointestinal cancer^63^. The pangenome analysis carried out in this study revealed 26 unique genes (Table S2) in L16 with four unique prophages. Although the functions of prophage genes are still enigmatic, they could contribute to the bacterial acquisition of novel genetic information^64^. Two sopB genes of L16 encode for the SopB proteins with suggested roles in *E. coli* F plasmid partitioning^65,66^.

In this study, AMR protein features of L16 were aligned to the eight closely related genomes (Fig. S3). As expected, the features were almost identical among the genomes except that L16 carries the chloramphenicol O-acetyltransferase. This enzyme is known to confer chloramphenicol resistance to pathogens via acetylation-mediated inactivation of the drug^67,68^. The findings from the analysis of the L16 genome demonstrate the great potential of WGS techniques in providing adequate and reliable data for surveillance and monitoring of antimicrobial resistance. However, the high cost and a lack of expertise in sequencing and bioinformatics data analysis are significant challenges in resource-limited countries. The phenotypic resistance pattern observed in the current study shows that conventional antimicrobial susceptibility testing complements the genotypically predicted resistance and molecular determinants. Hence, a combined approach will be the most appropriate for monitoring resistance. Such comprehensive investigations with an integrated approach are highly recommended to fully understand the AMR landscape and follow the dynamic virulence of the rapidly evolving pathogens in Bangladesh. The overall findings of the present study will provide helpful information to the local authorities towards the development of a robust antimicrobial stewardship program.

This study has several limitations. We investigated clinical samples from the Dhaka division of Bangladesh, which might not represent the overall situation in Bangladesh. A more extensive study including all the broader territories or divisions and a larger sample size is highly desirable to monitor the trends in resistance patterns over a time period. Determination of ESBL phenotypes could have been a valuable addition to this study from the epidemiological perspective as unexpressed ESBL genes in antibiotic-susceptible isolates may lead to horizontal gene transfer through such strains^69^. Also, screening more isolates for the presence of ARGs and WGS analysis and inclusion of ARGs for each class of antibiotics would provide better correlation between phenotypic resistance and molecular markers. The presence of mobile genetic elements and whether the resistance genes are plasmid-encoded may further be studied to ascertain the threats associated with the transmission of resistance.

## Conclusion

High resistance rate to multiple antibiotics including carbapenems combined with multiple ARGs and sequence type 131 in clinical isolates, as revealed in this study, suggests that the situation is alarming in Bangladesh where irrational use of antibiotics combined with inadequate AMR surveillance and facilities to detect MDR and ESBL genes are common. The findings of this study strongly support the urgent need for a robust, comprehensive, and regular antibiotic surveillance system and the development of rapid diagnostic services to guide antibiotic treatment to check further emergence of dreadful isolates like L16. The inclusion of modern techniques such as continuous WGS level molecular surveillance is highly desirable to track the spread of MDR pathogens in Bangladesh and fill the current gaps in the knowledge of the molecular mechanisms of resistance.

## Materials and methods

### Sampling and isolates

A total of 100 clinical samples including urine and sputum were collected from different diagnostic centers (Popular Diagnostic Center, Dhanmondi; Popular Diagnostic Center, Mirpur; and the diagnostic lab of Bangabandhu Sheikh Mujib Medical University) located in Dhaka city, Bangladesh during January 2019 to April 2019. The samples were diluted serially and plated on MacConkey agar and Eosin Methylene Blue (EMB) agar media. Single colonies with the typical characteristic appearance and green metallic sheen on EMB agar media were picked and subcultured for obtaining pure isolates. The isolates were subjected to biochemical characterization as per standard microbiological procedures^3^. *E. coli* colonies were tested for growth on triple sugar iron agar (TSI) and lysine iron agar (LIA) and citrate utilization, urease production, indol fermentation, glucose degradation (methyl red test), and motility. One isolate per sample was selected and a total of 100 isolates were stored in nutrient broth with 15% glycerol at − 80 °C.

### Antibiotic resistance profiling of the isolates

The isolates were subjected to antibiotic susceptibility tests against 12 antibiotics belonging to 10 different classes using Kirby-Bauer disk diffusion method in Mueller–Hinton agar media according to the guidelines of the Clinical and Laboratory Standard Institute^70^. The antimicrobial discs included were as follows: amoxycillin (10 µg), gentamycin (10 μg), ciprofloxacin (5 µg), norfloxacin (10 μg), cefuroxime (30 μg), imipenem (10 μg), meropenem (10 μg), chloramphenicol (30 μg), azithromycin (15 μg), tetracycline (30 μg), co-trimoxazole (25 μg) and piperacillin-tazobactam (100/10 μg). Results obtained were used to classify isolates as being resistant, intermediate resistant, or susceptible to a particular antibiotic using standard reference values according to Clinical and Laboratory Standards Institute^70^. *E. coli* strain ATCC 25922 was used as a reference. For each isolate, the multiple antibiotic resistance (MAR) index was calculated using the formula; MAR index = a/b, where a = number of isolates resistant to antibiotics and b = total number of antibiotics used. MDR (Multidrug-resistant) isolate was defined as the isolate showing resistance to three or more classes of antibiotics tested. Non-susceptibility to at least one agent in all but two or fewer antimicrobial categories were identified as XDR.

### Genomic DNA extraction and detection of antibiotic resistance genes

Genomic DNA was extracted from overnight grown bacterial cultures using a Genomic DNA purification kit (Promega, Wizard DNA Purification Kit) according to the manufacturer’s instructions. The integrity of DNA samples was checked by electrophoresis on 0.8% (w/v) agarose gel. The isolates were tested for the presence of antibiotic resistance genes by PCR using primer sets listed in Table 5. The assays were carried out in 20 µL reaction mix containing 1 µL of forward and reverse primer each (20 pmol/µL), 2 µL of template DNA, and 10 µL of Taq master mix (Favorgen, USA). The following conditions were used: initial denaturation at 94 °C for 5 min, 30 cycles of denaturation at 94 °C for 30 s, annealing at corresponding temperature (Table 5) for 45 s, extension at 72 °C for 1 min, and final extension at 72 °C for 7 min. The presence of a target gene was confirmed by the presence of a band on the agarose gel.

**Table 5:** Primers and PCR conditions for amplification of AMR genes.

Target gene	Primer sequence (5’–3’)	Annealing temperature (°C)	Amplicon size (bp)	References
*bla*_OXA-1_ group	F: ACACAATACATATCAACTTCGC R: AGTGTGTTTAGAATGGTGATC	56	814	^14^
*bla*_OXA-47_	F: TCAACTTTCAAGATCGCA R: GTGTGTTTAGAATGGTGA	47	609	^14^
*bla*_TEM_	F: TCGGGGAAATGTGCGCG R: TGCTTAATCAGTGAGGACCC	58	850	^14^
*bla*_CTX-M-15_	F: CACACGTGGAATTTAGGGACT R: GCCGTCTAAGGCGATAAACA	56	996	^14^
*bla*_SHV_	F: CACTCAAGGATGTATTGTG R: TTAGCGTTGCCAGTGCTCG	56	861	^14^
*tetC*	F: CTTGAGAGCCTTCAACCCAG R: ATGGTCGTCATCTACCTGCC	58	418	^71^
*bla*_NDM_	F: GGTGCATGCCCGGTGAAATC R: ATGCTGGCCTTGGGGAACG	56	660	^72^
*ampC*	F: TGAGTTAGGTTCGGTCAGCA R: AGTATTTTGTTGCGGGATCG	56	98	^72^

### Genome sequencing, assembly and annotation

Whole genome sequencing (WGS) was carried out for the isolate (L16) that showed resistance to all the antibiotics used in the study. Genomic DNA from the cultured isolate was prepared using a Qiagen Genomic-tip kit, and the sequencing was conducted on an Illumina MiSeq platform at the facilities of Genome Research Institute, NSU, followed by Fastqc and MultiQC analysis. The reads were then subjected to the comprehensive genome analysis service at PATRIC^20^, where the strain was designated. The genome was annotated using the RAST tool kit (RASTtk)^22^ and assigned a unique genome identifier. Subsystems of the genome were compared with two *Escherichia coli* reference genomes using PATRIC.

### In silico typing, AMR profile, virulence genes, and mobile genetic elements analysis

Sequence type (ST) was analyzed using the multilocus sequence typing (MLST) 2.0 tool^73^ from the Center for Genomic Epidemiology (CGE) webserver (https://cge.cbs.dtu.dk/). *E. coli* scheme 1 was selected, which employs seven housekeeping *E. coli* genes (adk, fumC, gyrB, icd, mdh, purA, recA) as proposed by Wirth et al. in 2006^74^. To determine the serotype of L16, we used SerotypeFinder 2.0^75^ from the CGE webserver. In silico phylotyping was performed by ClermonTyping^76^ which separates the isolates into 8 phylogroups (A, B1, B2, D, C, E, F, and cryptic clades) based on the presence or absence of 5 genes: chuA, yjaA, tspE4.C2, arpA and trpA. A k-mer-based AMR genes detection method was utilized in PATRIC’s genome annotation service, which assigned functional annotation to broad antibiotic resistance mechanisms. Isolate resistome was analyzed using the Resistance Gene Identifier (RGI) tool of the Comprehensive Antibiotic Resistance Database (CARD), where partial genes were excluded, and the predictions were made with contigs > 20,000 bp. Virulence genes were screened by VFDB, a reference database for bacterial virulence factors (http://cge.cbs.dtu.dk/services/VirulenceFinder/) that contains 139 virulence genes, including 44 ExPEC related genes^77,78^. Mobile genetic elements (MGEs) were predicted using the MobileElementFinder web tool^26^.

### Pangenome analysis

For pangenomic analysis, we downloaded 46 genome assemblies from NCBI (https://www.ncbi.nlm.nih.gov/assembly, accessed on May 02, 2021) with the search term “(562[Taxonomy ID]) AND Bangladesh”. The test isolate and the 46 assemblies were subjected to Prokka^79^ annotation and Roary pangenome pipeline^80^.

## Supplementary Information

Supplementary Information 1.

Supplementary Information 2.

Supplementary Information 3.
