# Supplementary material for: High prevalence of multiple antibiotic resistance in clinical E. coli isolates from Bangladesh and prediction of molecular resistance determinants using WGS of an XDR isolate
Source: Sci Rep. 2021 Nov 24;11:22859. doi: 10.1038/s41598-021-02251-w (PMC8613203; doi:10.1038/s41598-021-02251-w)
Supplement: Supplementary file 3 — Supplementary Information 3. [file 41598_2021_2251_MOESM3_ESM.pdf]

High prevalence of multiple antibiotic resistance in clinical *E. coli* isolates from Bangladesh and prediction of molecular resistance determinants using WGS of an XDR isolate

Preeti Jain[1], Asim Kumar Bepari[1], Prosengit Kumer Sen[1], Tanzir Rafe[1], Rashed Imtiaz[1], Maqsud Hossain[2,3], Hasan Mahmud Reza[1*]

[1]Department of Pharmaceutical Sciences, North South University, Dhaka, Bangladesh

[2]NSU Genome Research Institute (NGRI), North South University, Dhaka, Bangladesh

[3]Department of Biochemistry and Microbiology, North South University, Dhaka, Bangladesh

Table S1: Numbers of specialty genes in L16, K-12 substr. MG1655 and O157:H7 str. Sakai.

**Property**	**Source**	**K-12 substr. MG1655**	**O157:H7 str. Sakai**	**L16**
Antibiotic Resistance	CARD	75	77	84
	NDARO	1	1	6
	PATRIC	74	80	65
Drug Target	DrugBank	387	385	388
	TTD	58	60	59
Transporter	TCDB	892	909	874
Virulence Factor	PATRIC_VF	215	283	214
	VFDB	60	128	95
	Victors	230	297	229

Table S2: Unique genes in L16 compared to other 46 *E. coli* assemblies from Bangladesh

**Annotation**	**Number of Genes**	**Genes**
CP4-57 prophage; predicted antirestriction protein	2	*YfjX_3*, *yfjX_4*
DLP12 prophage; DNA packaging protein	1	*nohB_1*
DLP12 prophage; predicted murein endopeptidase	1	*rzpD_2*
DsdC DNA-binding transcriptional dual regulator	1	*dsdC_1*
Hypothetical protein	17	-
InsAB' transposase	1	*insAB-1*
IS1 protein InsA	1	*insA-1*
Protein SopB	2	*SopB_1*, *sopB_2*

Table S3: Eight public genomes closely related to L16.

**Genome Name**	**GenBank Accessions**	**Collection Date**	**Isolation Country**	**Host Name**
Escherichia coli strain Ecol_656	CP018979	2012	USA	Human
Escherichia coli strain Ecol_AZ146	CP018991	2012	Italy	Human
Escherichia coli strain 4/0	CP023849	2009	Sweden	Human
Escherichia coli strain S65EC	CP036245	2009	Australia	Human
Escherichia coli str. TO217	LS992192		Germany	-
Escherichia coli strain p11A	CP049077	2015	USA	Human
Escherichia coli strain p4A	CP049085	2015	USA	Human
Escherichia coli strain THO-003	AP022525	2018	Japan	Human

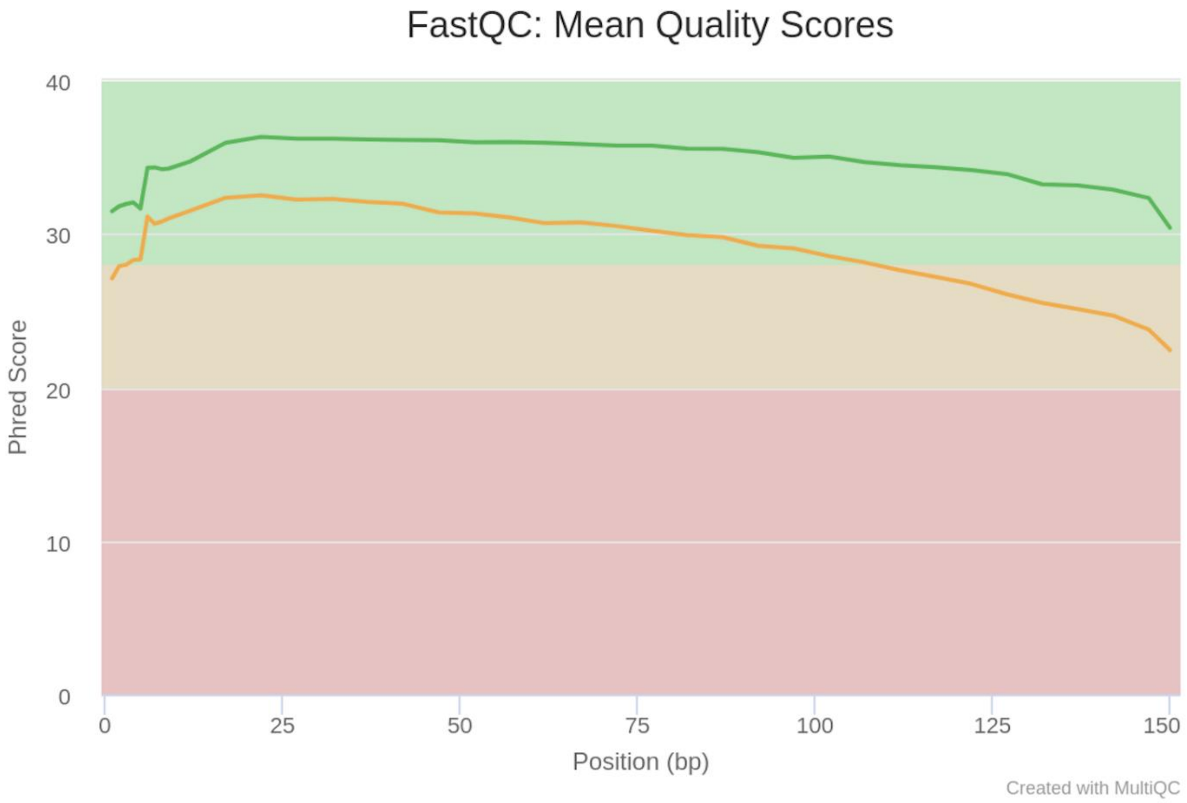

Figure S1: Mean quality values (Phred scores) of sequence reads. Green, forward reads; orange, reverse reads.

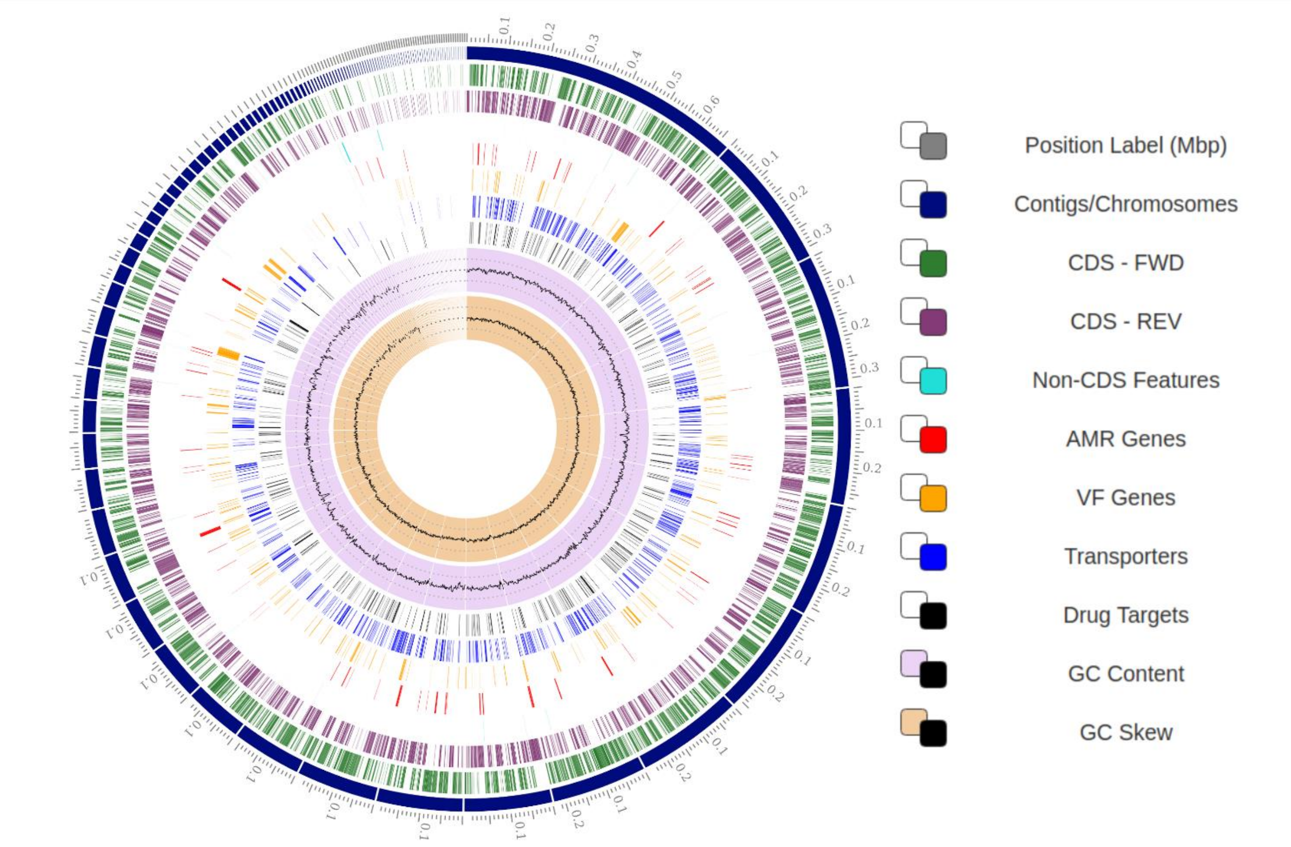

Figure S2: Circular view of the L16 genome.

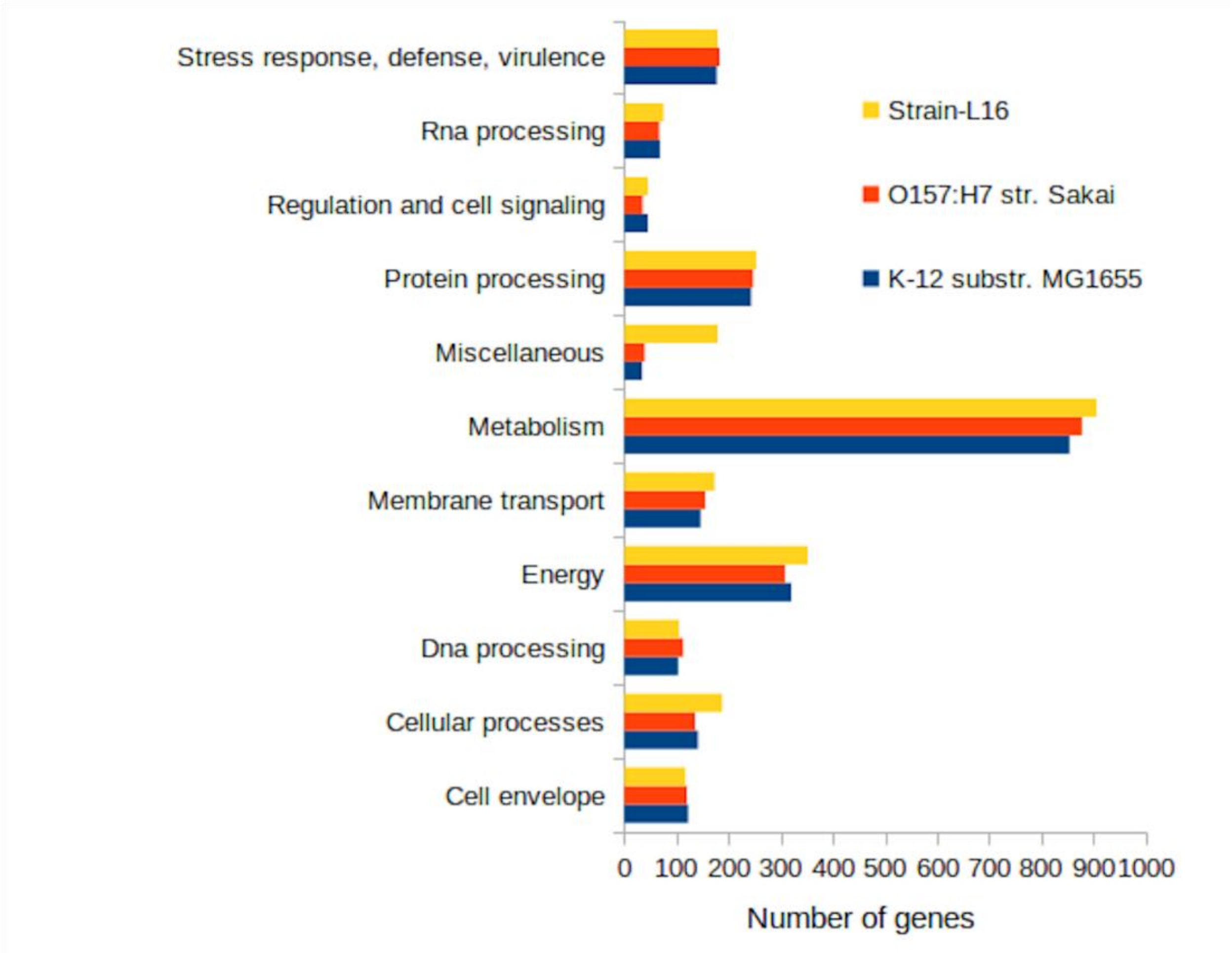

Figure S3: Comparison of the protein subsystems of L16 with two *E. coli* reference genomes, K-12 substr. MG1655 and O157:H7 str. Sakai.

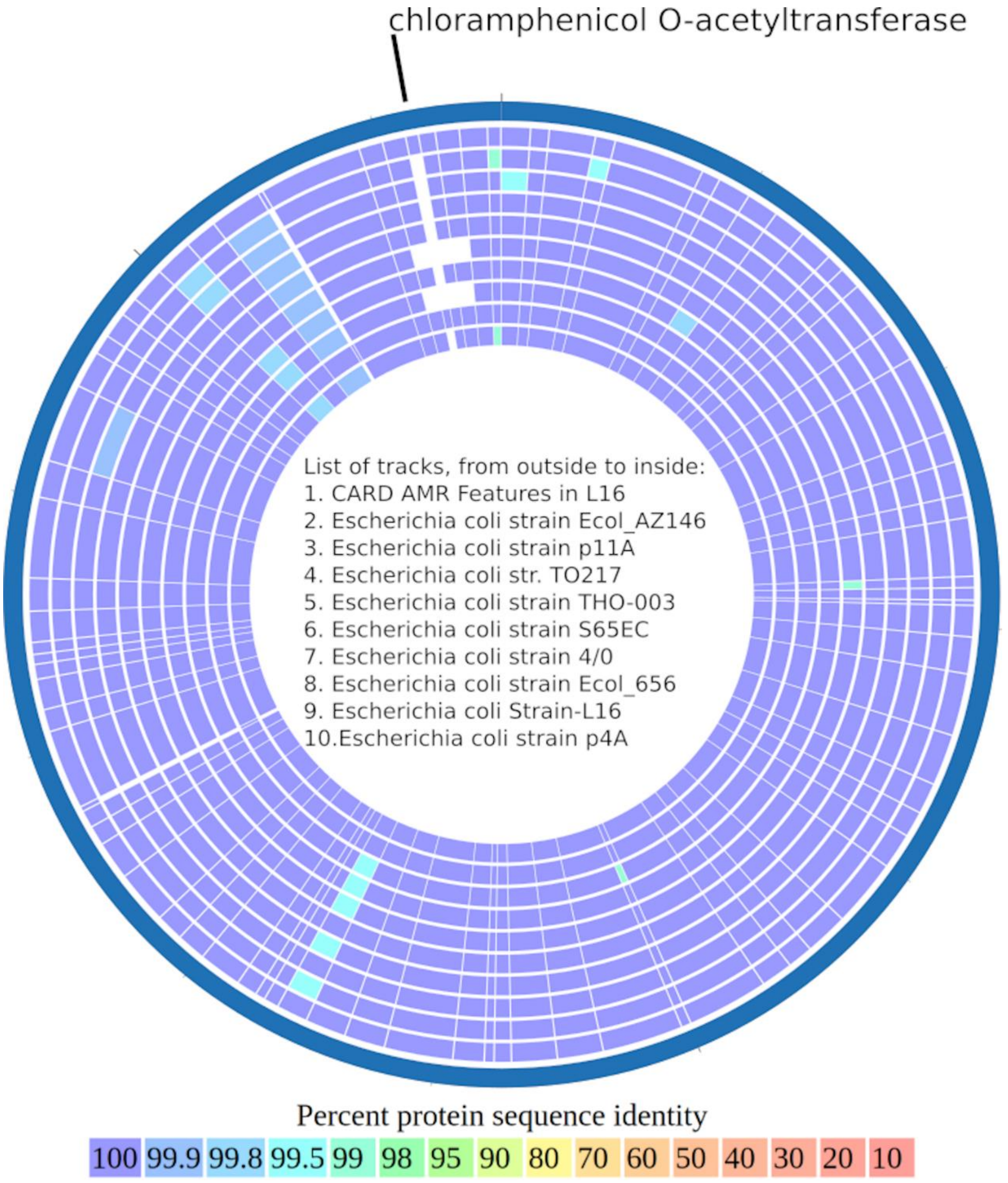

Figure S4: PATRIC alignment of the AMR protein features of L16 to the eight closely related genomes.
